# Transcranial Magnetic Stimulation with the Maximum Voluntary Muscle Contraction Facilitates Motor Neuron Excitability and Muscle Force

**DOI:** 10.1155/2012/847634

**Published:** 2012-03-20

**Authors:** Tetsuo Touge, Yoshiteru Urai, Kazuyo Ikeda, Kodai Kume, Kazushi Deguchi

**Affiliations:** ^1^Health Sciences, School of Nursing, Faculty of Medicine, Kagawa University, Kagawa 761-0793, Japan; ^2^Department of Neurology, Tokyo Metropolitan neurological Hospital, Tokyo, Japan; ^3^Gastroenterology and Neurology, School of Medicine, Faculty of Medicine, Kagawa University, Kagawa, Japan

## Abstract

Three trials of transcranial magnetic stimulation (TMS) during the maximum voluntary muscle contraction (MVC) were repeated at 15-minute intervals for 1 hour to examine the effects on motor evoked potentials (MEPs) in the digital muscles and pinching muscle force before and after 4 high-intensity TMSs (test 1 condition) or sham TMS (test 2 condition) with MVC. Under the placebo condition, real TMS with MVC was administered only before and 1 hour after the sham TMS with MVC. Magnetic stimulation at the foramen magnum level (FMS) with MVC was performed by the same protocol as that for the test 2 condition. As a result, MEP sizes in the digital muscles significantly increased after TMS with MVC under test conditions compared with the placebo conditions (*P* < 0.05). Pinching muscle force was significantly larger 45 minutes and 1 hour after TMS with MVC under the test conditions than under the placebo condition (*P* < 0.05). FMS significantly decreased MEP amplitudes 60 minutes after the sham TMS with MVC (*P* < 0.005). The present results suggest that intermittently repeated TMS with MVC facilitates motor neuron excitabilities and muscle force. However, further studies are needed to confirm the effects of TMS with MVC and its mechanism.

## 1. Introduction

Transcranial magnetic stimulation (TMS) is a noninvasive method of stimulating cortical neurons; that is, electrical currents in axons of interneurons stimulated by TMS activate cortical neuron cell bodies via synaptic transmission [1]. Single TMS or repetitive TMS (rTMS) can transiently inhibit or facilitate cortical neuron excitabilities for a prolonged period following stimulation [2–5]. According to these lines of evidence, many studies have tested whether TMSs or rTMS accelerates functional recovery in patients with motor disability [6–11].

A previous study reported that only three single TMS during the maximum voluntary muscle contraction (MVC) in patients with weakness of the thigh muscles transiently, but significantly, enhanced muscle strength compared with TMS during muscle relaxation [12]. Nevertheless, the effects of TMS with MVC have not yet been established, and its mechanism still remains unknown. In the present study, we investigated the effects of TMS with MVC on motor neuron excitability by recording motor evoked potentials (MEPs) with MVC, using a modified protocol of TMS with MVC to induce more prolonged and robust effects on motor neuron function. Furthermore, we stimulated the corticospinal tract at the foramen magnum level to detect the functional mechanism of TMS with MVC.

Preliminary results of the present study were previously reported in the third International Conference on Complex Medical Engineering (CME2009) on April 9–11, 2009 in Tempe, Arizona.

## 2. Subjects and Methods

This study was approved by the local ethics committee of our institution. All subjects who were healthy volunteers consented to participate in the present study after receiving an explanation on the protocol and safety of the experiments.

### 2.1. TMS with MVC Procedure

Nine subjects including seven females and two males (age range: 21–45 years, mean age ± S.D.: 28.9 ± 10.4 years) participated in the study. Subjects sat on a comfortable chair and totally relaxed their voluntary muscles. They performed MVC by pinching a button-like strain-gauge transducer measuring 1.5 cm diameter (9E01-L2, NEC San-ei, Japan) using the right thumb and index finger with maximum force. To induce maximum force of the right first dorsal interossei muscle (FDI), the transducer was pressed on the left side of the distal interphalangeal joint of the index finger by the thumb. Each MVC trial for 2 seconds was started and stopped quickly responding to verbal cues by the operator. TMS was delivered using a round coil of 10 cm diameter connected to a single pulse magnetic stimulator (SMN-1200, Nihon-Kohden, Japan). Stimulus intensity was 150% of the active motor threshold in the right FDI. We defined the active motor threshold as the intensity to induce MEPs in right FDI with amplitudes greater than 50 *μ*V in at least 50% of successive TMS trials during sustained pinching of the strain-gauge transducer by the right thumb and index finger with maximum muscle force [13]. TMS was delivered 1 second after subjects started MVC, in which timing muscle force approximately reached the top, and TMS was repeated 4 times with an interstimulus interval of 10 s.

### 2.2. Recording of MEPs and Pinching Muscle Force

**Figure 1:**
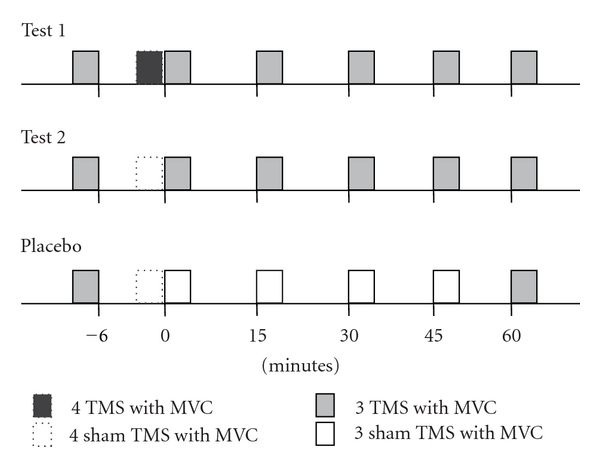
The time schedule of TMS with MVC under the test 1, test 2, or placebo conditions. MEPs were recorded at time points marked as 3 TMSs with MVC.

To induce MEPs using Ag/AgCl surface electrodes in FDI and the thenar muscle (TH), the left motor cortex for FDI and TH was stimulated using the apparatus described above with a stimulus intensity of 110% of the active motor threshold of the right FDI. TMS was delivered 1 second after subjects started MVC for 2 seconds responding to the operator's verbal cues. One session of MEP recording consisted of 3 TMS trials with an interstimulus interval of 10 s. MEPs were recorded for six sessions: 5 minutes before and just after the 4 trials of TMS with MVC, and each 15 minutes for 1 hour after the trials under test 1 condition (Figure 1). The pressure for pinching the strain-gauge transducer was simultaneously recorded as electric signals during MVC as a marker of the muscle force of FDI and TH, and the signals for pressure were converted to Kg as that 10 Kg was 0.11 V.

Under the test 2 condition, sham TMS was delivered in the 4 trials of TMS with MVC with the round coil positioned tangentially and its lower edge fixed on the vertex. Other experimental settings were the same as those for the test 1 condition (Figure 1). Furthermore, we recorded 3 MEPs 5 minutes before and 1 hour after the 4 trials of sham TMS with MVC under the placebo condition. At other time points for MEP recording under the test conditions, sham TMS was delivered during 3 MVC trials. Recording of MEPs and pinching muscle force was performed by 8 subjects under each condition. However, pinching muscle force was recorded in only 6 subjects under the test 1 or 2 condition due to mechanical problems.

### 2.3. Foramen Magnum Stimulation

Two of the nine subjects and novel seven subjects including 2 males and 7 females (age range: 18–45 years, mean ± S.D.: 24.6 ± 10.0 years) participated in the study. TMS at the foramen magnum level was delivered using an eight-figure coil with the maximum output of the apparatus according to the method in the study by Ugawa et al. [14]. The lower edge of the coil was initially placed at the foramen magnum level and was then moved to the optimal point to induce MEPs in the right FDI. Other experimental settings were similar to those in the test 2 condition.

### 2.4. Data Analysis

Amplitudes and areas of three MEPs were measured and averaged off line at each recording session. Voltages and latencies from baselines to peaks of pressure signals induced via the strain-gauge transducer during 3 MVC were measured and averaged. Pinching muscle forces were converted to ratios to those in the first session of TMS with MVC. Data were statistically analyzed using repeated measure of analysis of variance (ANOVA) and Dunnett post hoc analysis (SPSS Ver. 17). In ANOVA, F values were corrected by Greenhouse-Geisser *ε* (GGE) if necessary.

## 3. Results

MEP amplitudes or areas of FDI and TH before TMS with MVC were similar among the three conditions, and pinching muscle force before TMS with MVC did not significantly differ among the groups (Table 1).

### 3.1. Changes of MEP Sizes by TMS with MVC

**Figure 2:**
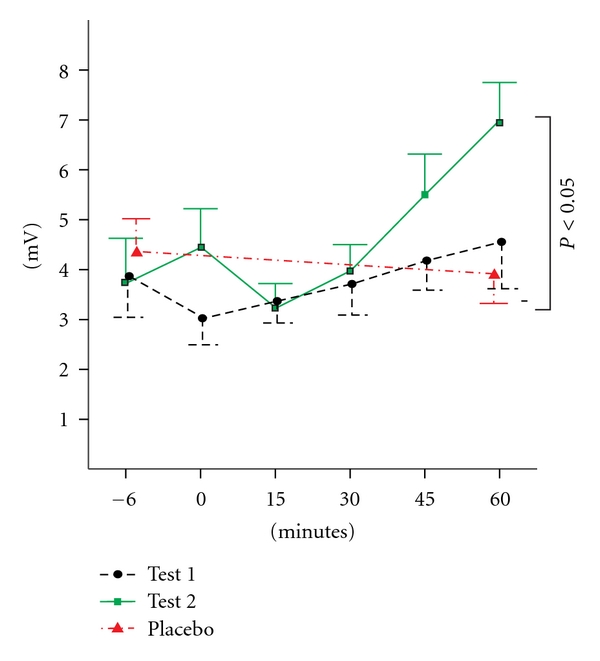
Changes of MEP amplitudes of FDI before and after TMS with MVC under the test 1, test 2, or placebo conditions. Values show means ± S.E.

Changes in MEP amplitudes in FDI after TMS with MVC compared with those before TMS with MVC significantly differed among the three conditions (*F*2, 21 = 4.662, GGE = 1.0, *P* < 0.05) (Figure 2). Changes in MEP areas of FDI and MEP amplitudes or areas of TH were similar among the conditions. There were significant differences in the changes of MEP amplitudes (*F*1, 14 = 25.85, *P* < 0.001) or areas (*F*1, 14 = 4.181, *P* < 0.02) in FDI after TMS with MVC between the test 2 and the placebo conditions. The test 1 condition had significant differences in changes of MEP amplitudes in TH after TMS with MVC than the placebo condition (*F*1, 14 = 5.63, *P* < 0.05).

**Figure 3:**
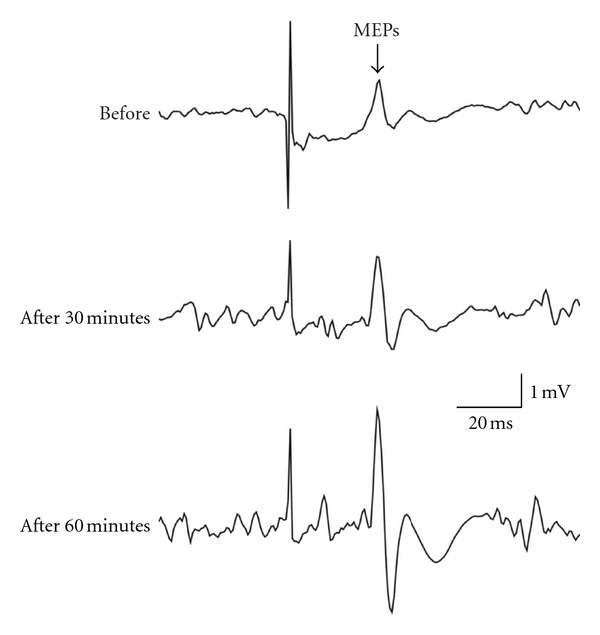
A sample of changes in MEPs of FDI muscle during TMS with MVC under the test 2 condition.

Under the test 2 condition, Dunnett's post hoc analysis showed that MEP amplitudes in FDI significantly increased 45 minutes (5.52 ± 0.82 mV, *P* < 0.05, mean ± S.E.) and 1 hour (7.03 ± 0.73 mV, *P* < 0.001) after TMS with MVC than before it (3.74 ± 0.90 mV) (Figure 3), and MEP areas in FDI were significantly larger 45 minutes (13.23 ± 2.88 mV×ms, *P* < 0.01) and 1 hour (13.77 ± 2.38 mV×ms, *P* < 0.01) after TMS with MVC than before it (7.59 ± 1.21 mV×ms). MEP amplitudes in TH significantly increased 30 minutes (5.32 ± 1.23 mV, *P* < 0.05) after TMS with MVC compared with those before TMS with MVC (3.13 ± 0.72 mV). The test 1 condition, showed no significant difference in MEP sizes from the test 2 condition. There was no significant change in MEP sizes under the test 1 condition which tended to slightly decrease just after TMS with MVC and increase 60 minutes after TMS with MVC.

### 3.2. Changes of Pinching Muscle Force by TMS with MVC

**Figure 4:**
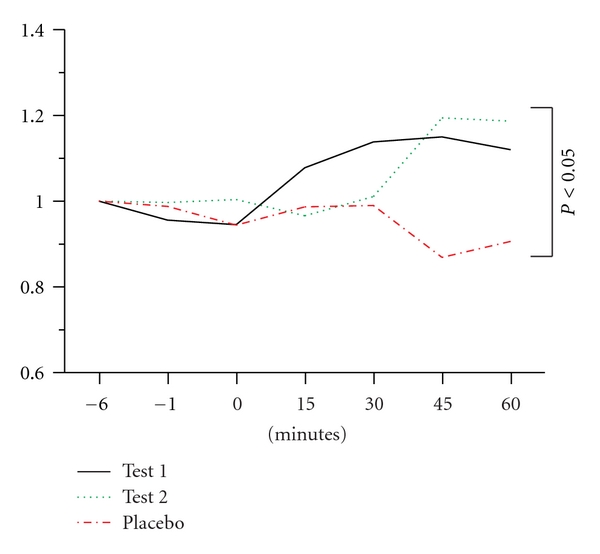
Changes in the ratios of the maximum pinching muscle force before and after TMS with MVC under the test 1, test 2 or placebo condition. Values show averaged ratios to the maximum pinching force in the first session of TMS with MVC.

Changes in pinching muscle force by TMS with MVC showed significant differences among the three conditions (*F*12, 102 = 2.140, GGE = 0.603, *P* < 0.05) (Figure 4). The test 1 or 2 condition showed a significant difference in changes in muscle force by TMS with MVC compared to those under the placebo condition (*F*6, 72 = 2.428, *P* < 0.05 in the test 1 condition or =3.265, *P* < 0.01 in the test 2 condition). The data at 45 and 60 minutes after TMS with MVC under the test 1 condition (1.14 ± 0.11 and 1.12 ± 0.04, resp.) or the test 2 condition (1.19 ± 0.12 and 1.19 ± 0.07, resp.) were significantly larger compared with those in the placebo condition (0.87 ± 0.06 and 0.91 ± 0.07, resp., *P* < 0.05).

### 3.3. Changes of MEP Sizes and/or Pinching Muscle Forcer by Foramen Magnum Stimulation with MVC

**Figure 5:**
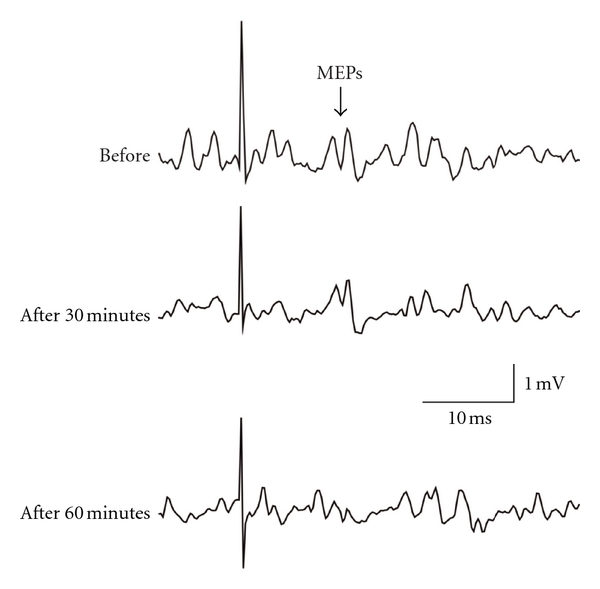
A sample of changes in MEPs of FDI during foramen magnum stimulation with MVC under the test 2 condition.

Changes in MEP amplitudes in TH or FDI by foramen magnum stimulation showed a significant interaction with those in the test 2 condition (*F*1, 5 = 6.35, *P* < 0.001 in TH and *F*1, 5 = 8.38, *P* < 0.001 in FDI). Changes in MEP areas in FDI by foramen magnum stimulation significantly differed from those in the test 2 condition (*F*1, 5 = 4.46, *P* < 0.005). Dunnett's post hoc analysis showed that MEP amplitudes in FDI were significantly decreased 60 minutes after the stimulation with MVC (1.80 ± 0.48 mV, *P* < 0.05) than before it (1.03 ± 0.23 mV) (Figure 5). MEP areas, TH amplitudes, and TH areas were not significantly changed by foramen magnum stimulation with MVC. Pinching muscle force did not significantly change by foramen magnum stimulation with MVC.

## 4. Discussion

The present study showed that TMS with MVC transiently increased MEP sizes and pinching muscle force. This result supports a previous report that documented enhancing MVC force and voluntary activation (VA) of the quadriceps femoris muscles by TMS with MVC in normal subjects [12]. Furthermore, we demonstrated novel findings that intermittently repeated TMS with MVC significantly increased MEP amplitudes and muscle force compared with the placebo condition.

We stimulated the motor cortex by TMS with a stimulus frequency (SF) of 0.1 Hz. A previous study showed that single TMS repeated at intervals of 10 seconds did not have any prolonged effect on cortical motor neuron excitabilities [2]. However, the dual stimulation of TMS with an interstimulus interval of 10 seconds and persistent peripheral nerve or motor point stimulation, known as paired associative stimulation (PAS), had facilitative effects on MEP amplitudes as well as inducing prolongation of the silent period and expansion of muscle representation on the scalp [15, 16]. Since F wave elicited by peripheral nerve stimulation and MEPs induced by electrical stimulation of the brain stem were not affected by PAS, the effective point of this technique was suggested to be in the cortical neurons. The mechanisms like long-term potentiation (LPT) of synaptic transmission were speculated as the mechanisms for PAS [17]. Another study demonstrated the fact that intracortical facilitation at a short interstimulus interval (0.8–2.0 ms), assessed by the paired TMS technique, was enhanced just after PAS, and which was considered to reflect I wave interaction within the motor cortex [18].

Prolonged peripheral nerve stimulation induced reorganization of the cortical motoneurons, as increasing corticospinal outputs or changing cortical representation of the stimulated muscles [18, 19]. Median nerve stimulation, in which trains for 1 millisecond with an SF of 10 Hz were delivered at an SF of 1 Hz for 2 hours, increased muscle force in stroke patients [20]. In the present study, intermittently repeated MVC during 1 hour performed by the subjects continuously activated muscle afferents, and repetitive muscle afferent inputs appear to facilitate sensorimotor integration in the sensory and motor cortices as observed in prolonged peripheral nerve stimulation [18, 19]. In addition, TMS delivered synchronously during brisk thumb movements enhanced motor memory encoding of thumb movements [21]. This finding appears to coordinate with the Hebbian principle that LTP of synaptic transmission is induced when pre- and postsynaptic fibers are simultaneously activated [22]. Considering these findings, the facilitative effects of TMS with MVC on motor neuron function appear to share a mechanism similar to that of PAS. Since TMS with low stimulus intensity principally activates presynaptic interneurons in the cortex, synaptic efficacy of cortical motoneurons seems to be increased by TMS with MVC [11, 12, 23].

Impulses in corticospinal tract fibers generated by foramen magnum stimulation are transmitted to anterior horn cells in the spinal cord. Such impulses appear to activate facilitatory and/or inhibitory interneurons in contact with synaptic terminals of the upper motor neurons or anterior horn cells [23]. Additionally, magnetic impulses delivered with high stimulus intensity (SI) spread and activate the neck and occipitofrontal muscles, and afferent inputs from those muscles most likely influence activities of the anterior horn cells. Single TMS to the C6/7 nerve root inhibited MEPs by cortical stimulation lasting for some 5 ms [24]. Furthermore, rTMS at 1 Hz with subthreshold SI to the right posterior neck facilitated MEPs in hand muscles elicited by TMS and Hoffman reflex [25]. On the other hand, spread of magnetic currents with the suprathreshold SI may stimulate the cerebellum despite the use of a figure-eight coil for foramen magnum stimulation. Many previous studies have shown that single TMS or rTMS to the cerebellum can modulate cortical motor neuron excitabilities [26]. However, magnetic stimulation at the foramen magnum level with a suprathreshold SI during MVC has not previously been investigated. The present study showed a novel finding that foramen magnum stimulation with MVC applied intermittently for 1 hour significantly decreased MEP amplitudes in FDI but did not change pinching muscle force. Therefore, the decreased MEP amplitudes by foramen magnum stimulation with MVC suggest inhibition of spinal motor neuron excitabilities, while the facilitative effects of TMS with MVC on motor neuron excitabilities are ascribed to the supraspinal mechanism.

 The test 1 condition showed increases of MEP amplitudes in TH after TMS with MVC compared with the placebo condition. In addition, the test 1 condition tended to be decreased MEP sizes just after application of TMS with MVC, although the changes of MEP sizes did not reach statistical significances. In the experiment, TMS was delivered with high SI, 1.5 times higher than the active motor threshold, and could induce almost the maximum MEP amplitudes. Previous studies showed that sustained or repeated MVC induces fatigue as expressed by reduced muscle force and subsequent increment of MEP amplitudes as a result of supraspinal fatigue [27, 28]. We presume that high-intensity TMS with MVC is capable of inducing fatigue in the muscles or spinal cord just after TMS application and may have masked the increment of muscle force and MEP amplitudes, which would explain why there were no significant differences in the changes in MEP sizes between the test conditions. We did not find any evidence of supraspinal fatigue in the present study because we employed a brisk and short MVC as a task.

In conclusion, we consider that intermittently repeated TMS with MVC facilitates motor neuron function and is applicable to accelerating functional recovery of motor disability caused by an impaired central nervous system. However, further studies are needed to confirm the effects of TMS with MVC and elucidate details of the mechanism.

## Figures and Tables

**Table 1:** MEP sizes and pinching muscle force before TMS with MVC under each condition.

		MEP amplitudes (mV)		MEP areas (mV × ms)		Pinching muscle force (V)
		FDI	TH	FDI	TH	
Conditions	Test 1	3.88 ± 0.85	2.31± 0.36	11.64 ± 3.85	6.69 ± 1.45	13.2 ± 2.0
	Test 2	3.74 ± 0.90	3.13 ± 0.72	7.59 ± 1.21	8.62 ± 2.26	14.5 ± 2.2
	Placebo	4.37 ± 0.67	3.64 ± 0.82	12.40 ± 2.84	10.77 ± 2.91	18.3 ± 2.6

Data are shown as mean ± S.E. There were 8 data sets except for pinching muscle force under the test 1 and test 2 conditions (*n* = 6). MEP: motor evoked potentials, TMS: transcranial magnetic stimulation, MVC: the maximum voluntary muscle contraction, FDI: the digital interossei muscle, TH: the thenar muscle.
